# Insecticidal and growth inhibitory potential of *Streptomyces hydrogenans* DH16 on major pest of India, S*podoptera litura* (Fab.) (Lepidoptera: Noctuidae)

**DOI:** 10.1186/s12866-014-0227-1

**Published:** 2014-08-28

**Authors:** Talwinder Kaur, Arti Vasudev, Satwinder Kaur Sohal, Rajesh Kumari Manhas

**Affiliations:** Department of Microbiology, Guru Nanak Dev University, Amritsar, 143005 Punjab India; Insect Physiology Lab, Department of Zoology, Guru Nanak Dev University, Amritsar, 143005 Punjab India

**Keywords:** *Streptomyces hydrogenans*, *Spodoptera litura*, Larvicidal, Nutritional assay, Growth inhibitory

## Abstract

**Background:**

Destructive impacts of insecticides on non targeted populations necessitate the development of an eco friendly pest control method. *Streptomyces* spp*.* are rich source of bioactive secondary metabolites which may provide valuable alternatives to chemical insect-control agents as they can be less toxic and readily biodegradable. Because of its potent biocontrol attributes, ethyl acetate extract of *Streptomyces hydrogenans* DH16, a soil isolate, was tested to assess its anti-insect potential against polyphagous noctuid, *Spodoptera litura*.

**Results:**

The secondary metabolites in the ethyl acetate extract of *S. hydrogenans* DH16 exhibited larvicidal and growth inhibitory activities. The results indicated that highest concentration of 1600 μg/ml was significantly effective as 70% larval, 66.66% prepupal and 100% pupal mortality was noticed. The metabolites also prolonged the larval developmental period. The LC50 and LC90 values were 1337.384 and 2070.516 μg/ml, respectively for the insect*.* Negative effects of *S. hydrogenans* were also observed on development of the insect. Significant decline in adult emergence, adult longevity, fecundity and % hatching was recorded at higher concentrations along with morphological abnormalities as compared to control. Significant decrease in relative growth and consumption rate, efficiency of ingested and digested food and increase in approximate digestibility in larvae reared on diet supplemented with ethyl acetate extract accounts for the toxic as well as anti-nutritive nature of extract.

**Conclusion:**

Secondary metabolites in the fermentation broth from *S. hydrogenans* were toxic to the larvae at higher concentrations whereas lower concentrations significantly reduced the reproductive potential of *S. litura*. Therefore, these metabolites show considerable potential for incorporation in pest management programmes as new biopesticidal formulation.

## Background

According to the report of FAO, due to the attack from pathogenic organisms and insect pests, 20–40% decrease in crop yield occurs which results in loss of 120 billion US $ worldwide [1]. Pest insects, being plant disease vectors reduce crop output by 10–30% either by reducing the quality and quantity of the crop production, or by serving as vectors of plant diseases [2]. *Spodoptera litura* (Fabricius) (Lepidoptera: Noctuidae), a polyphagous insect of cosmopolitan distribution, has a large host range of more than 150 host species [3] and is considered economically important in many countries including India, Japan, China and Southeast Asia [4]. This defoliating insect pest affects the yield of various cultivated crops, vegetables, weeds and ornamental plants by feeding gregariously on leaves and causes large economic losses of crop plants. It was reported as a major pest in groundnut in Andhra Pradesh, India and caused 28–100% yield loss depending upon crop stage and its level of infestation [5,6]. The management of *S. litura* to ensure the stable and high output of crops is a great challenge in agricultural field and therefore, insecticide use is most widely practiced for its control. However, there is widespread concern over negative impact of insecticides on environmental and human health due to accumulation of insecticide residues as well as emergence of pesticide resistance in the pests [7]. Application of chemical pesticides also kills different varieties of pest predators and results in ecological imbalance, thereby causing pest resurgence and a greater outbreak of secondary pests [8]. Therefore, there is a need for developing safe and eco-friendly alternatives to chemical insecticides for pest control.

Biological control as a part of integrated pest management has gained interest among researchers as it is an environmentally friendly and a safe strategy for pest management [9]. Natural products obtained from plants and microorganisms have been used for insect control [10]. Azadirachtin (complex limonoids), a natural compound isolated from Indian neem tree, *Azadirachta indica* A. Juss (Meliaceae), is known to have lethal effects on more than 400 insect species [11] and many workers have used azadirachtin as positive control [12–14]. Recently, microbial insecticides have attracted considerable attention [15] because they are more specific, have low relative cost and are more eco-friendly [16–18]. Among the biological control agents derived from different microbes, actinobacteria especially *Streptomyces* spp. are one of the most important microbial resources which can provide potential new bioactive compounds for use as insect-control agents [19]. Many reports indicated the important role played by actinobacteria in the management of S*podopetra littoralis* (Biosduval) [20], *S. litura* [21], *Musca domestica* (Linnaeus) [22], *Culex quinquefasciatus* (Say) [23], *Drosophila melanogaster* (Meigen) [24], *Helicoverpa armigera* (Hubner) [25], *Anopheles* mosquito larvae [26]. Bream et al. [20] showed potent biological activity of secondary metabolites of actinobacteria such as *Streptomyces* and *Streptoverticillum* against *S. littoralis* which caused larval and pupal mortality. Several metabolites from genus *Streptomyces,* such as avermectin, prasinons, doramectin, milbemycin, nanchangmycin, dianemycin and spinosad have been established as potential protective agents against a variety of pest insects and are friendly to environment [27,28]. In light of this and inspired by the remarkable pharmaceutical and agricultural potential of bioactive metabolites of actinobacteria, Kaur et al. [29] screened actinobacterial isolates, recovered from different rhizospheric and non-rhizospheric soils, for antifungal activity against fungal phytopathogens and reported strong insecticidal activity against *S. litura* in one of the isolates, *Streptomyces hydrogenans* DH16 which also exhibited potent antifungal activity [30]. Present study was aimed at further systematic evaluation of antifeedant, larvicidal, pupicidal and growth inhibitory effect of solvent extract from *S. hydrogenans* DH16 against *S. litura*.

## Results and discussion

There is a long history of utilizing natural products produced by microbes for pharmaceutical and agricultural purposes. Actinobacteria especially, *Streptomyces* spp. have provided wide variety of secondary metabolites of high commercial importance and continue to be routinely screened for new bioactive compounds. Present work further corroborates the earlier findings and reports that secondary metabolites from *S. hydrogenans* exhibit the potential to be used as insecticidal agents. In this study, *S. hydrogenans* extract showed deleterious effects on growth and development of *S. litura* larvae that survived the toxic effects of highest concentration. Significant increase in larval development period was observed at all concentrations over the control (P ≤ 0.05). At highest concentration (1600 μg/ml), larval period prolonged by 6.24 days in comparison to control group (Table 1). Our result coincided with the findings of Arasu et al. [21] who reported larvicidal and growth inhibitory activities of a novel polyketide metabolite isolated from *Streptomyces* sp. AP-123 against *H. armigera* and *S. litura.* The metabolite also prolonged the larval–pupal duration of the insects at all the tested concentrations as compared to control. The delayed larval period observed in the present study could be due to low consumption of diet by the larvae of *S. litura* indicating the antifeedant effect of the extract. Pupal period decreased significantly with treatment (P ≤ 0.01) however, at highest concentration pupae formed from treated larvae remained in pupal stage till the termination of experiment. The total development period from larva to adult of *S. litura* differed but remained non significant (Table 1). The LC50 and LC90 values were 1337.384 and 2070.516 μg/ml, respectively for *S. litura* (Table 2). No larval mortality was observed in lowest concentration as well as in control but when larvae were fed on highest concentrations of 800 and 1600 μg/ml, larval mortality of 20 and 70%, respectively was recorded and was statistically significant compared to control (P ≤ 0.01). Similar study on soil bacterium metabolite 12-epi-Hapalindole J isonitrile isolated from *Cyanobacterium terium* showed 100% larval mortality against dipteran *Chironomus riparius* (Meigen) at 26 μM concentration within 48 h of exposure [31]. Similarly, anti-insect activity of crude ethanolic extracts from *Streptomyces* sp. in terms of larval mortality had been reported by Rishikesh et al. [32]. The isolate showed a marked insecticidal activity against *Sitophilus oryzae* in a dose dependent manner with 100% mortality at concentration of 24 mg/ml. Later, Arasu et al. [21] documented 68.41% and 60.02% larvicidal activities by polyketide metabolite from *Streptomyces* sp. AP-123 against *H. armigera* and *S. litura*, respectively at 1000 ppm. Azadirachtin showed a more toxic effect towards *S. litura* as compared to the crude extract of *S. hydrogenans* as 100% mortality was noticed at higher concentrations.

**Table 1:** **Influence of ethyl acetate extract of** ***S. hydrogenans*** **on and azadirachtin on various developmental parameters of** ***S.litura***

**Treatments**	**Concentrations (μg/ml)**	**Larval period (in days) (Mean ± S.E.)**	**Pupal period (in days) (Mean ± S.E.)**	**Total developmental period (in days) (Mean ± S.E.)**
*Streptomyces* ethyl acetate extract	400	17.30 ± 0.19^ab^	10.36 ± 0.40^ab^	27.66 ± 0.40
	800	19.97 ± 2.15^ab^	8.03 ± 0.76^b^	28.00 ± 0.93
	1600	22.00 ± 2.11^b^	-	-
	f- value	3.30*	5.83**	0.62^N.S^
	R^2^	0.99	0.82	0.57
Azadirachtin	400	16.66 ± 0.33^c^	7.00 ± 0.36^c^	-
	800	-	-	-
	1600	-	-	-
	f- value	-	-	-
	R^2^	-	-	-

Mean ± SE followed by different letters (superscript) with in a column are significantly different. Tukey’s test P ≤ 0.05, N.S = Non Significant, R^2^ = Coefficient of determination, *Significant at 5% level, **Significant at 1% level.

**Table 2:** **Regression equation, lower as well as upper 95% confidence limits for LC**_**50**_ **and LC**_**90**_

	**Regression equation**	**95% Confidence limit**		**LC**_**50**_	**LC**_**90**_
		**Lower**	**Upper**	**(μg/ml)**	**(μg/ml)**
*Streptomyces* ethyl acetate extract		1164.962^a^	1562.021^a^	1337.384	2070.516
	Y = 6.751X-16.107	1729.403^b^	2989.165^b^		
		32.516^c^	363.252^c^	260.121	560.390
Azadirachtin	Y = 3.866X-9.344	427.265^d^	1142.37^d^		

^a^Lower and upper 95% confidence limits for LC_50_ for *Streptomyces* ethyl acetate extract, ^b^Lower and upper 95% confidence limits for LC_90_ *Streptomyces* ethyl acetate extract, ^c^Lower and upper 95% confidence limits for LC_50_ for azadirachtin, ^d^Lower and upper 95% confidence limits for LC_90_ for azadirachtin.

**Figure 1:**
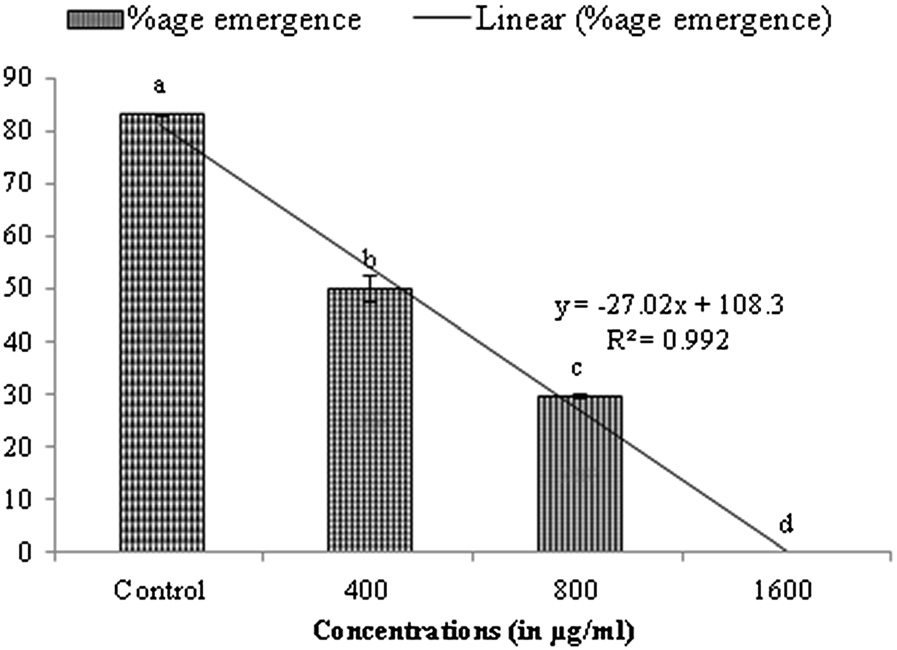
**Effect of ethyl acetate extract of** ***S. hydrogenans*** **on % age emergence of** ***S.litura.*** Columns and bars represent the mean ± SE. Different letters above the columns representing each concentration indicate significant differences at Tukey’s test P ≤ 0.05.

Prepupal mortality (66.66%) was also higher at the highest concentration (P ≤ 0.01) (Table 3). Diet supplemented with extract of *S. hydrogenans* induced 48–100% pupal mortality. As compared to control, significantly higher mortality of more than 50% was recorded at highest concentrations (P ≤ 0.01) (Table 3). Similarly, dose dependent (125–1000 ppm) pupal mortality (18–62%) was reported by Arasu et al. [21] and documented that prolonged larval–pupal durations were directly proportional to the increase in pupicidal activities. The adverse effect of solvent extract was also observed on emergence and performance of adults emerged from treated larvae. Adult emergence was significantly lower when larvae were reared on diet amended with extract (P ≤ 0.01) and the decrease was found to increase with increase in concentration (Figure 1). Maximum adverse effect was observed at highest concentration where no adult emergence occurred. Also, adults emerged at lower concentrations were small in size with varied abnormalities. Xiong et al. [33] found that out of 40 isolates from marine micro-organisms, *Streptomyces* sp.173, similar to avermectin B1 possessed strong insecticidal potential against *H. armigera*. In another study, Xiong et al. [34] reported strong inhibitory activity of *Streptomyces avermitilis* strain 173 isolated from marine source against *Heliothis zea* (Boddie), *Plutella xylostella* (Linnaeus), *Spodoptera exigua* (Hübner) and aphids.

**Table 3:** **Effect of ethyl acetate extract of** ***S. hydrogenans*** **and azadirachtin on mortality rate of different developmental stages of** ***S.litura***

**Treatments**	**Concentrations (μg/ml)**	**Larval mortality (%)**	**Prepupal mortality (%)**	**Pupal mortality (%)**	**Corrected Pupal mortality (%)**
	Control	-	-	13.80 ± 0.67^a^	-
*Streptomyces* ethyl acetate extract	400	-	-	48.26 ± 1.01^b^	39.98 ± 1.40^a^
	800	20.00 ± 00.00^a^	20.00 ± 4.47^a^	57.13 ± 2.09^c^	50.26 ± 0.45^b^
	1600	70.00 ± 12.40^b^	66.66 ± 0.38^b^	100.00 ± 00^d^	100.00 ± 0.00^c^
	f- value	16.30**	107.79**	863.97**	1436.26**
	R^2^	0.80	0.81	0.94	0.94
Azadirachtin	400	76.66 ± 1.59^c^	-	85.70 ± 1.22^e^	83.41 ± 0.45^d^
	800	96.66 ± 0.42^d^	-	-	-
	1600	100.00 ± 00^e^	-	-	-
	f- value	146.19**	-	-	-
	R^2^	0.85	-	-	-

Mean ± SE followed by different letters with in a column are significantly different. Tukey’s test P ≤ 0.05, R^2^ = Coefficient of determination, **Significant at 1% level.

Adult survival time was also influenced by the *S. hydrogenans* as longevity of emerged adults declined significantly from 11.50 days in control to 4.33 days at 800 μg/ml (P ≤ 0.01) (Table 4). Fecundity in emerged adults from treated larvae was also significantly inhibited. It declined from 1500 eggs/female (control) to 150.20 eggs/female at 400 μg/ml concentration (P ≤ 0.01). The viability of these eggs was also negatively affected as the eggs failed to hatch whereas in control 87.66% hatching of eggs was observed (Table 4). No egg laying was recorded at 800 μg/ml concentration. Abouelghar et al. [35] also demonstrated the negative effects of sublethal concentrations of spinosad on development, fecundity and food utilization in the cotton leafworm, *S. littoralis* (Boisd.).

**Table 4:** **Effect of ethyl acetate extract** ***S. hydrogenans*** **on longevity, fecundity and percent hatching of** ***S.litura*** **adults**

**Concentrations (μg/ml)**	**Longevity (in days) (Mean ± S.E.)**	**Fecundity (No. of eggs laid/ female) (Mean ± S.E.)**	**Percent Hatching (Mean ± S.E.)**
Control	11.50 ± 0.76^a^	1500 ± 151.00^a^	87.66 ± 0.91
400	5.00 ± 0.77^b^	150.20 ± 22.40^b^	-
800	4.33 ± 0.66^b^	-	-
1600	-	-	-
f- value	28.89**	78.64**	-
R^2^	0.91	0.67	0.60

Mean ± SE followed by different letters with in a column are significantly different. Tukey’s test P ≤ 0.05, R^2^ = Coefficient of determination, **Significant at 1% level.

### Morphological abnormalities

**Figure 2:**
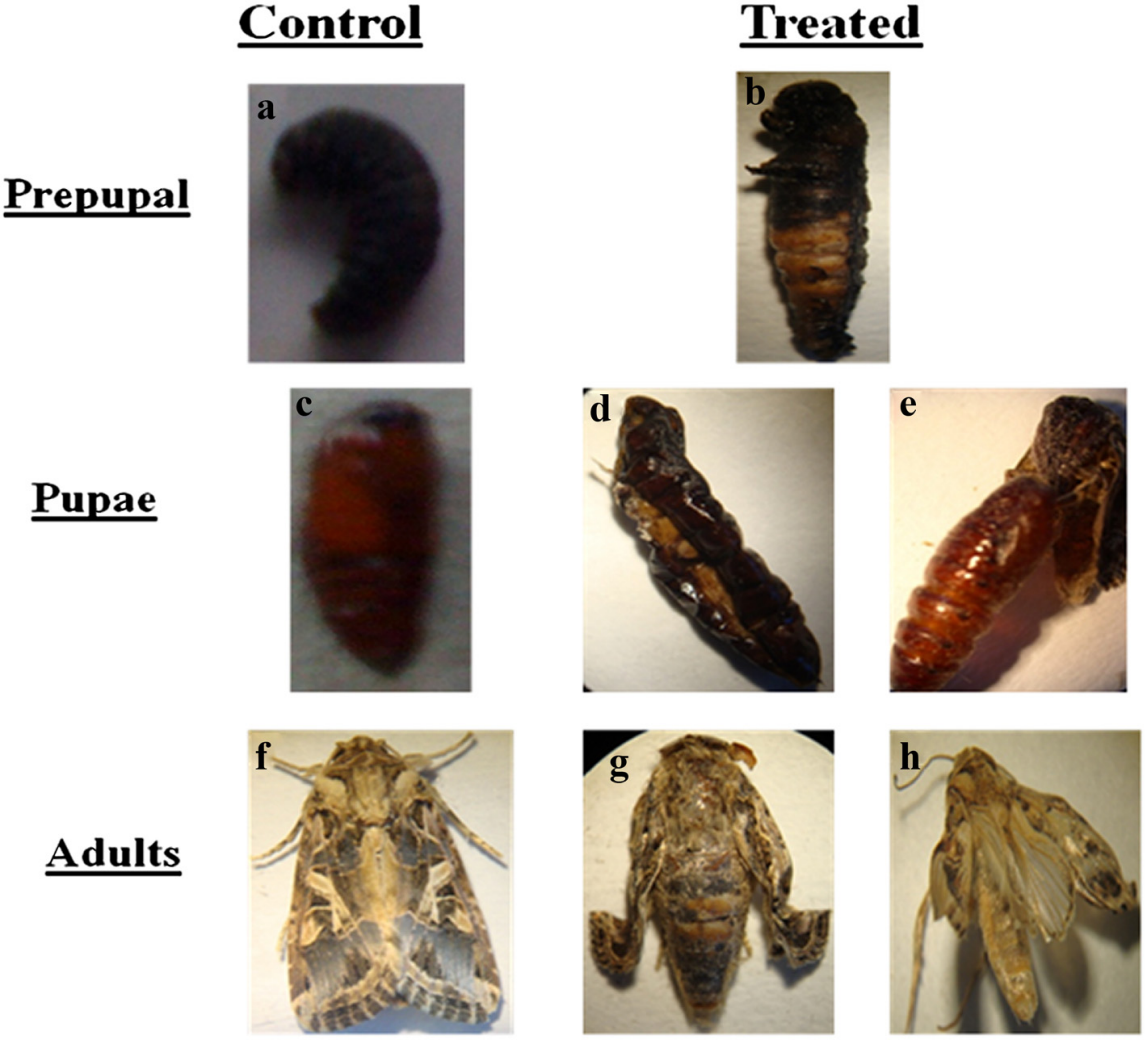
**Developmental stages of** ***S.litura*** **reared on control diet (a,c,f) and abnormalities in different stages fed on diet supplemented with different concentrations of ethyl acetate extract of S. hydrogenans (b,d,e,g,h).**

The inhibitory effect of extract was further manifested in the form of deformed adults which emerged from the larvae fed on *S. hydrogenans* extract supplemented diet. The deformed adults had crumpled and underdeveloped wings as well as were half emerged from pupa. These deformities in adults were recorded only at 400 and 800 μg/ml concentrations (Figure 2).

### Food utilization assay

The diet utilization experiments indicated significant effect of *S. hydrogenans* solvent extract on *S. litura*. As is apparent from Table 5, there was significant decrease in relative growth and consumption rate of *S. litura* as well as efficiency of conversion of ingested and digested food. Diet supplemented with extract resulted in 13–49% reduction in RGR over the control (P ≤ 0.01). Food consumption rate reduced to half of that in control at highest concentration (P ≤ 0.01).

**Table 5:** **Effect of ethyl acetate extract of** ***S. hydrogenans*** **and azadirachtin on food utilization and feeding of** ***S.litura***

**Treatments**	**Concentrations (μg/ml)**	**RGR (mg/mg/day) (Mean ± S.E.)**	**RCR (mg/mg/day) (Mean ± S.E.)**	**AD (%) (Mean ± S.E.)**
	Control	2.17 ± 0.07^a^	6.97 ± 0.39^a^	28.35 ± 1.05^a^
*Streptomyces* ethyl acetate extract	400	1.88 ± 0.03^ab^	7.29 ± 0.26^a^	30.00 ± 0.29^a^
	800	1.66 ± 0.10^b^	6.99 ± 0.38^a^	51.96 ± 0.44^b^
	1600	1.10 ± 0.11^c^	3.53 ± 0.29^b^	66.00 ± 1.33^c^
	f- value	26.45**	27.53**	416.91**
	R^2^	0.95	0.59	0.92
Azadirachtin	400	1.54 ± 0.20^d^	3.92 ± 0.80^c^	43.56 ± 9.37^d^
	800	-	-	-
	1600	-	-	-
	f- value	-	-	-
	R^2^	-	-	-

Mean ± SE followed by different letters with in a column are significantly different. Tukey’s test P ≤ 0.05, R^2^ = Coefficient of determination, *Significant at 5% level, **Significant at 1% level.

**Figure 3:**
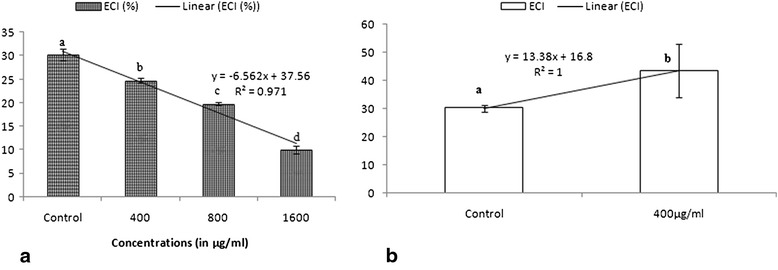
**Effect of (a) ethyl acetate extract of** ***S. hydrogenans*** **and (b) Azadirachtin on ECI of** ***S.litura*** **.** Columns and bars represent the mean ± SE. Different letters above the columns representing each concentration indicate significant differences at Tukey’s test P ≤ 0.05.

**Figure 4:**
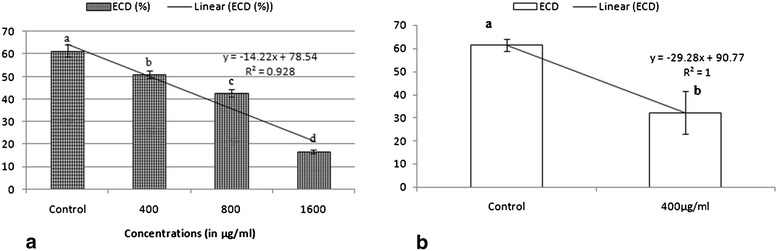
**Effect of (a) ethyl acetate extract of** ***S. hydrogenans*** **and (b) Azadirachtin on ECD of** ***S.litura*** **.** Columns and bars represent the mean ± SE. Different letters above the columns representing each concentration indicate significant differences at Tukey’s test P ≤ 0.05.

A concentration dependent decrease in ECI and ECD was observed in the larvae of *S. litura* (Figures 3 and 4). The diet amended with extract caused 18–67% decline in ECI and 17–72% decline in ECD over the control. Approximate digestibility increased by 43% at 1600 μg/ml in comparison to control as shown in Table 5 (P ≤ 0.01). The reduction in diet utilization suggests that reduced growth and development might have resulted from both behavioral and physiological effects. It is likely that this decrease in consumption rate (RCR) could be due to the antifeedant nature of the extract and accounts for the majority of the decrease in growth rate (RGR). The *Streptomyces* extract also altered food utilization indices in *S. litura* and revealed less conversion of ingested (ECI) and digested (ECD) food to body biomass. The extract also influenced AD of larvae fed on amended diet as it increased with increase in concentration but the increase in AD could not compensate for the decrease in ECD, which consequently led to reduced growth rate. ECI is an overall measure of an insect’s ability to utilize the food that it ingests for growth and development and ECD is a measure of the efficiency of conversion of digested food into growth [36]. A drop in ECI indicates more food is being metabolized for energy purpose and less for conversion to body substance. ECD also decreases as the proportion of digested food metabolized for energy increases. Thus, decreased ECI and ECD values in the present studies indicate that ingested crude extract of *Streptomyces* does exhibit some chronic toxicity against *S. litura* [37].

## Conclusions

Present study reports growth inhibitory activities of metabolites of *S. hydrogenans* on *S. litura*. The metabolites in the extract showed strong antifeedant, larvicidal, pupicidal and toxic activities against major pest *S. litura*. Diet utilization experiments clearly revealed the growth inhibitory impact of extract. However, the toxic effect of the extract was less as compared to the positive control, azadirachtin, which could be due to the purified nature of the plant compound. These findings indicate that the extract has considerable potential to control insect pest populations and can further be used for development of novel insecticidal formulation as an alternative to toxic chemicals for the management of field pests.

## Methods

*Streptomyces hydrogenans* DH16 (GenBank: JX123130) was isolated from soil, procured from Dalhousie, Himachal Pradesh, India and identified using polyphasic taxonomic approach [29]. Culture was maintained on starch casein nitrate agar (SCNA, starch: 10 g/l, NaCl: 2 g/l, KNO_3_: 2 g/l, K_2_HPO_4_: 2 g/l, CaCO_3_: 0.02 g/l, MgSO_4_: 0.05 g/l, FeSO_4_: 0.01 g/l, casein: 0.3 g/l and agar: 20 g/l) slopes at 4°C and as mycelial fragments and spores in 20% v/v glycerol at −80°C.

### Production and extraction of bioactive metabolites from *Streptomyces*

Production and extraction of solvent extract of *S. hydrogenans* was carried out by the method of Kaur and Manhas [29].The isolate was cultured on starch casein nitrate agar medium at 28°C. After 7 days of incubation, the growth was scrapped and transferred aseptically into the seed medium (SCN broth) and incubated for 48 h to develop inoculum. The seed culture, at a concentration of 2%, was inoculated into production medium having same composition as seed medium and fermentation was carried out at 28°C at 180 rpm for optimum production of bioactive metabolites. After three days, the flasks were harvested and the biomass was separated from the culture broth by centrifugation at 10000 rpm for 20 min at 4°C. After centrifugation, the active metabolites in the cell free fermented broth were extracted in ethyl acetate and organic phase was concentrated under vacuum to yield a brown colored extract which was re-dissolved in dimethyl sulfoxide (DMSO) and was stored at 4°C for further use.

### Insect culture

*S. litura* is a widely spread species and is found in much of the Asia and Oceania regions [3]. For rearing, egg masses of *S. litura* were collected from cauliflower planted in the fields around Guru Nanak Dev University, Amritsar (Punjab), India. The culture was maintained in the B.O.D. incubator at a temperature of 27 ± 2°C, relative humidity 60% and photoperiod (L16:D18) on castor (*Ricinus communis*) leaves in battery jars (l15 × d10 cm). The leaves were washed with sodium hypochlorite solution (1%) and changed regularly till pupation. The pupae were separated and kept in pupation jars provided with moist sterilized sand. After adult emergence, adult moths were transferred to oviposition jars in the ratio of 1 male: 2 females and covered with muslin cloth. The jars, containing cotton soaked in 20% sugar solution, were lined with filter paper to aid egg laying. The eggs were kept in small Petri plates having a moist cotton swab. After hatching, the larvae were fed on artificial diet (bran: 6 g, kidney bean flour: 30 g, yeast extract: 3 g, agar: 3 g, vegetable oil: 375 μl, streptomycin: 0.3 g, vitamin-B complex: 0.6 g, formaldehyde: 600 μl and distilled water 195 ml) [12]. Bran, kidney bean flour, vegetable oil and formaldehyde were mixed together. Agar was boiled separately in 100 ml of distilled water in beaker. The dissolved agar was poured into the above said mixture and stirred for 4–5 mins. Rest of the diet contents were added at last to the mixture and mixed thoroughly. The whole diet was poured into sterilized Petri plates while still hot. The diet was allowed to cool at room temperature for 24 h and stored at 4°C before giving to larvae. Control diet was prepared without extract and treated diet had different concentrations of the extract.

### Bioassay studies

Bioassay studies were carried out to evaluate the effect of ethyl acetate extract from *S. hydrogenans* on growth and development of *S. litura*. For this, the artificial diet was supplemented with three concentrations (400, 800 and 1600 μg/ml) of extract as well as respective controls. Then, 2^nd^ instar (5 to 6 days old) larvae were starved for 2–3 h and transferred individually to plastic containers (49 × 6 cm) containing cubical pieces of treated and control diets. The experimental trays were kept in B.O.D incubator maintained under controlled conditions and observed daily for various parameters such as larval period, pupation time, number of pupae formed, the number of adults emerged, fecundity and percent hatching. Larval, prepupal and pupal mortality was also recorded. The diet was changed regularly. Each experiment was replicated six times with 5 larvae/replication (n = 120). Abbott’s formula was used to correct mortality in the control group (only for % pupal mortality) as given below:

$$ \frac{\mathrm{Mt} - \mathrm{Mc}\ }{100 - \mathrm{Mc}}\times \kern0.5em 100 $$

Where Mt: % age mortality in treated group, Mc: % age mortality in control group

For the fecundity assay, ten pairs of moths that emerged on the same day from control and 2–3 pairs from treatment group were collected and put into a battery jar lined with filter paper to facilitate egg laying and absorbent cotton soaked in a 10% sugar solution was provided for moth nutrition. The egg-masses laid were counted daily under stereomicroscope (Magnüs, 10X) and removed individually to a petri dish for further observation. To evaluate the fertility, egg-masses obtained from control and treatment group were observed daily for hatching, and then the hatch percent was calculated.

### Nutritional indices

The nutritional indices of *S. litura* were determined by following the procedure of Koul et al. [38]. To find out weight gain, food consumption and feces produced, gravimetric technique was used. All weights were measured in milligrams (mg) using a monopan balance (Citizen) accurate to 0.1 mg. Newly molted 2^nd^ instar larvae were starved for 1–2 h to clear their digestive tracts. After measuring the initial weight of the larvae carefully with the help of brushes, they were individually introduced into experimental plastic containers containing weighed quantities of control and treated diet. The larvae (30 larvae/concentration including control, 6 replicates) were allowed to feed for a period of three days on diet supplemented with extract as well as control. After this feeding period, larvae were again weighed and weights of larvae, uneaten diet and faecal matter were taken at the end of the experiment. The net gain or loss in terms of body weight (wet) of individual larvae, food ingested by larva and fecal matter of larvae were calculated by subtracting the initial weight from the final weight at the end of experiment. Dry weights of larvae were taken by incubating the larvae at the end of experiments at 60°C for 72 h inside an incubator. Similarly dry weights of different samples of diet and faecal matter were also taken. The dry weight readings indicate water loss under control conditions. From the results the following nutritional indices were obtained as proposed by Waldbauer [39] and all indices were calculated using dry weights.

RGR and RCR were calculated on dry weight basis after 3 days of feeding as G/I (G = change in larval dry weight/day and I = starting larval dry weight) and C/I (C = change in diet dry weight/day and I = starting larval dry weight), respectively. Both were calculated as mg/mg/day. Index of food conversion efficiency (ECI) was calculated as 100 × G/C; where G = dry weight gain of insect and C = dry weight of food consumed. Approximate digestibility (AD) and efficiency of conversion of digested food (ECD) were calculated as C - F/C × 100 (where C = change in diet dry weight/day and F = dry weight of frass/day) and G/C-F × 100 (where G = change in larval dry weight/day, C = change in diet dry weight/day and F = dry weight of frass/day, respectively. ECI, AD and ECD were calculated as percent.

### Statistical analysis

Data collected from the above experiments were subjected to statistical analysis where values were represented as their mean ± SE. To compare difference in means, one way analysis of variance (ANOVA) was performed using Minitab (version 14), Tukey’s *post hoc* test was done with the help of ASSISTAT (7.7 beta). Linear regression analysis was performed to know coefficient of determination (R^2^) with Microsoft office excel 2007 (Microsoft Corp., USA). To calculate LC_50_ SPSS software for windows version 16.0 (SPSS Inc., Chicago) was used.
